# Questions Persist: Environmental Factors in Autoimmune Disease

**DOI:** 10.1289/ehp.119-a248

**Published:** 2011-06

**Authors:** Charles W. Schmidt

**Affiliations:** **Charles W. Schmidt**, MS, an award-winning science writer from Portland, ME, has written for *Discover Magazine*, *Science*, and *Nature Medicine*.

After his first child was born, black and blue marks started showing up on Stanley Finger’s body. Jolted awake most nights by his crying infant, Finger would stumble half asleep toward her room, bumping into walls and furniture in the dark. “My wife and I would joke about it,” says Finger, a chemical engineer from Bluffton, South Carolina. But during a routine checkup, Finger learned his easy bruising was caused by a precipitous drop in blood platelets. The body relies on these cell fragments for clotting, and Finger’s platelet count had dropped to nearly a third its normal value. After ruling out cancer and other illnesses, Finger’s doctor eventually arrived at a diagnosis: immune thrombocytopenia purpura (ITP).

ITP is an autoimmune disease, a condition that occurs when the immune system attacks the body’s own cells and tissues. When Finger was diagnosed in 1974, autoimmune illnesses weren’t yet perceived as the public health menaces they’re often seen as today. But according to Fred Miller, director of the Environmental Autoimmunity Group at the National Institute of Environmental Health Sciences, autoimmune diseases are now recognized as among the leading causes of death among young and middle-aged women in the United States.

What’s more, prevalence rates for some of these illnesses are rising for what Miller says must largely be environmental reasons. “Our gene sequences aren’t changing fast enough to account for the increases,” Miller says. “Yet our environment is—we’ve got 80,000 chemicals approved for use in commerce, but we know very little about their immune effects. Our lifestyles are also different than they were a few decades ago, and we’re eating more processed food.” Should prevalence rates for heart disease and cancer continue their decline, Miller says, autoimmune diseases could become some of the costliest and most burdensome illnesses in the United States.

## Sprawling Family of Illnesses

Until recently, scientists didn’t think of autoimmune illnesses as a group of related conditions. Instead, each illness was viewed as a unique and usually rare affliction. ITP, for instance, strikes barely 10 people of every 100,000^1^ (by comparison, the National Organization for Rare Disorders defines rare illnesses as those occurring in at most 250 people per 100,000 in the population). And since no one had tallied autoimmune diseases under a single umbrella, their cumulative impact on health and society wasn’t known.

That troubled Noel Rose, an immunologist and professor at The Johns Hopkins University, who was convinced that medical science wasn’t giving enough thought to what autoimmune diseases share in common. So in the mid-1990s, Rose began doing what no one else had done. Along with a small group of colleagues, he combed through published journal articles and other epidemiologic sources in an attempt to calculate how many people were afflicted with each of 24 autoimmune conditions, including multiple sclerosis, rheumatoid arthritis (RA), systemic lupus erythematosus (SLE), and type 1 diabetes mellitus.

Using 1996 population projections from the U.S. Census Bureau, Rose ultimately calculated these conditions, as a group, affected up to 1 in every 31 Americans—more than 8.5 million people at that time.^2^ “That was remarkable,” Rose says. “Until then, no one had realized that autoimmune diseases are so common.”

According to current estimates by the National Institutes of Health, as many as 23.5 million Americans may be afflicted with at least one autoimmune condition.^3^ But Rose says that number doesn’t account for 2010 U.S. Census data, and moreover, it’s drawn from the 24 autoimmune diseases considered in Rose’s assessment, when the actual number of these illnesses ranges from 80 to as many as 120.^4^ The actual size of the affected population in the United States could be as high as 50 million people,^5^ according to the American Autoimmune Related Diseases Association (AARDA), an advocacy group in East Detroit.

Autoimmune diseases tend to cluster among family members. Finger’s first child, for instance, has autoimmune hepatitis, while her sister has type 1 diabetes. However, even though identical twins have the same genetic susceptibility to inherited illness, Rose says it’s possible only one will develop an autoimmune condition, suggesting the involvement of environmental triggers.

Scientists define those triggers broadly: chemicals, infectious agents, stress, hormones, drugs, diet, weight gain, behavior, and more have all been cited as etiological factors. Rose acknowledges that changes in diagnosis might account for some of the increase. “That’s hard to rule out because there’s a lot more clinical awareness of these diseases now than there used to be,” he says. “But there are also some very good studies that show real increases, particularly for type 1 diabetes.”^6^,^7^ Given that type 1 diabetes has been well characterized for decades, this offers compelling evidence to date that rising incidence is not simply an illusion based on better diagnosis.

Jill Norris, a professor of epidemiology at the Colorado School of Public Health, adds that the prevalence of celiac disease—an autoimmune affliction of the small intestine triggered by exposure to gluten, a protein found in wheat, barley, and rye—also appears to be rising dramatically in the United States.^8^ “We’re probably seeing a mix of different trends,” Norris says. Where some autoimmune diseases are increasing, others are declining—for instance, the prevalence of RA appears to be falling in the population, she says. “And for most others,” she adds, “we don’t know, mainly because we don’t have the proper registries for tracking them.”

## Autoimmune Complexity Revealed

Scientists split autoimmune diseases into two general categories: organ-specific varieties (such as type 1 diabetes, which is an immune attack on insulin-producing cells in the pancreas) and systemic varieties (such as SLE, which occurs when the immune system turns against multiple organs and tissues throughout the body). Clinical outcomes vary by illness, ranging from bleeding disorders in ITP, to an inability to process glucose in type 1 diabetes, to joint pain and inflammation in RA.

Given that autoimmune diseases are often rare and may not be easily diagnosed with blood tests, imaging, and other standard tests, clinicians who aren’t familiar with these illnesses can find them perplexing. It’s not unusual for truly sick patients to be dismissed by medical professionals as lazy or neurotic, says Finger, now chairman of the AARDA board of directors. “It can take years to diagnose some of these conditions,” he says. “Patients can be shunted from one specialist to another. Even as late as 2000, mental health professionals were often the first to make a correct diagnosis.”

All autoimmune diseases occur when the body’s immune system turns against itself. But according to Kathleen Gilbert, an immunologist and professor at the University of Arkansas for Medical Sciences, that’s about the only thing we know for sure about them. Scientists have barely scratched the surface when it comes to knowing what triggers an autoimmune illness, she says.

The field is replete with competing theories and biological mechanisms, but it lacks a unifying concept, adds K. Michael Pollard, an associate professor at The Scripps Research Institute. “For every immunologist you’ll get one or two theories about what causes autoimmune disease,” he says. “That’s the state of the field—we’ve produced a lot of good work, but there are so many types of disease, and they all have mechanistic differences that might be subtle or they might be profound.”

In some cases, autoimmune illnesses occur when rogue proteins called autoantibodies target cells and tissues instead of foreign invaders like viruses and bacteria. That’s what happens in Graves disease—autoantibodies bind with hormone receptors on the thyroid gland. As a result, the organ becomes overactivated, leading to symptoms that include heat intolerance, unexplained weight loss, bulging eyeballs, hypertension, and tremor. Alternatively, T cells can target cells and tissues, Gilbert says, as occurs when they destroy insulin-producing islet cells in the pancreas, leading to type 1 diabetes.

But even T cell–mediated illnesses can be accompanied by a proliferation of autoantibodies that can be detected in blood before symptoms manifest. For instance, clinicians can assess autoimmune status among children thought to be at risk for type 1 diabetes by measuring blood levels of autoantibodies against insulin and other antigens.

Rose adds that some autoimmune diseases share heritable components, such as variations in the gene that codes for human leukocyte antigen (HLA). HLA is the human version of a major gene family called the major histocompatibility complex (MHC), which plays crucial roles in immunity in vertebrates. One MHC variation in particular, he says—known as the HLA-A1-B8-DR3 haplotype—is implicated in several autoimmune diseases. Indeed, most autoimmune illnesses can be tied to HLA variations of one kind or another, he adds. “So this tells me they’re fundamental to autoimmune disease etiology,” Rose says.

Still, Rose acknowledges that although many gene variations are linked to autoimmune diseases, each contributes just a small percentage to the overall risk. Disease occurs only when multiple genes act together, he says, and even then, genetics can’t explain the risk entirely, indicating that environmental factors are at play. “However, we have very little information about these factors,” Rose adds. “We need more data associating autoimmune illness with specific exposures. And we also need plausible biological mechanisms to explain how those exposures produce or exacerbate disease. This will dominate our research agenda over the course of the next decade.”

## Environmental Links

According to Pollard, the best evidence linking environmental exposures to autoimmune diseases so far comes from pharmaceutical drug studies. That’s not surprising, he says, given that patients in such studies are closely monitored with respect to dose, clinical outcome, and confounding from other factors. Two drugs in particular have been linked conclusively to SLE in a minority of patients, Pollard says: procainamide, a treatment for cardiac arrhythmia, and hydralazine, used for high blood pressure.^9^ “And when you take patients off the drugs, their lupus symptoms go away,” Pollard says.

Apart from pharmaceutical exposures and SLE, solid evidence also links gluten ingestion with celiac disease; indeed symptoms disappear upon gluten’s removal from the diet, according to Alessio Fasano, director of the Center for Celiac Research at the University of Maryland School of Medicine. However, human evidence for other environment–disease links is more tenuous, in part because of inherent limitations in environmental epidemiology, Pollard says: people tend to be mobile, they’re exposed to many environmental agents at once, and there’s often a significant time delay before the onset of autoimmune symptoms.

Proposed links also tend to suffer from conflicting study results. For instance, cigarette smoking was linked to SLE in studies from the United Kingdom, Sweden, and Japan,^10^,^11^,^12^ but three U.S.-based studies failed to show a similar connection.^13^,^14^,^15^

Sources interviewed for this story unanimously agree that rising prevalence rates are most evident in type 1 diabetes. Data from Finland, tracked by that country’s national health system, show type 1 diabetes rates more than doubled from 31 cases per 100,000 people in 1980 to 64 cases per 100,000 in 2005.^16^ Increases were also detected in 17 other European countries, at an average annual rise of 3.9% from 1989 to 2003.^17^ The authors of that study predicted the number of new cases in children younger than 5 years in Europe will double by 2020 compared with 2005, while the number of cases among those under age 15 will rise by 70%.^17^

Norris says increases in type 1 diabetes also have been documented in the United States through a program called SEARCH for Diabetes in Youth, which is coordinated by the U.S. Centers for Disease Control and Prevention.^18^ SEARCH tracks incidence data gathered by registries in six states—Colorado, California, Hawaii, Ohio, South Carolina, and Washington. Norris says cases in Colorado rose from 15 per 100,000 people in the 1978–1988 time frame to 23.9 per 100,000 between 2002 and 2004. “That’s about a 70% increase,” she says. “Not as big as what you’re seeing in Finland but substantial.”

## Diabetes: A Case in Point

What’s causing type 1 diabetes cases to rise? Terence Wilkin, a professor of endocrinology and metabolism at Peninsula Medical School in Plymouth, UK, cites steady increases in childhood weight at decreasing ages. According to Wilkin, heavier body mass exacerbates insulin resistance, or the process by which insulin becomes unable to coordinate glucose metabolism. That puts pancreatic beta cells into overdrive—as they struggle to meet insulin demands, the beta cells wear out, which initiates disease. “We consistently see that heavier children develop diabetes earlier in life,” Wilkin says. “And the link with insulin resistance explains why this is happening.”

Norris says Wilkin’s hypothesis makes some intuitive sense. But citing prospective data^19^ from the Diabetes Autoimmunity Study in the Young (DAISY), coordinated by the University of Colorado Denver, Norris says some children do show evidence of autoimmunity (measured by pancreatic islet cell autoantibodies in blood) before showing signs of being heavier or larger than normal. “In our data there is no association between weight or body mass index and future appearance of autoimmunity,” she explains. “It could be that weight stress and insulin resistance follow the initiation of autoimmunity,” she adds. “So, I wouldn’t entirely discount Wilkin’s theory—we might just have to tweak it to fit the data.”

Moreover, the DAISY cohort is limited to at-risk children identified by HLA type, Norris adds, whereas Wilkin’s hypothesis may apply to broader childhood populations that aren’t limited to genetically susceptible individuals. “And we haven’t tested Wilkin’s hypothesis in the general population,” Norris says.

Meanwhile, some investigators have proposed that type 1 diabetes might be related to consumption of infant formula, which became popular after World War II. Retrospective studies conducted mainly in Finland and other countries took that view one step further, showing associations with cow’s milk specifically.^20^ But more recent prospective investigations^21^,^22^,^23^,^24^—which Norris says aren’t biased by the recall problems that sometimes characterize retrospective designs—have been unable to confirm these associations. Instead, these studies have linked type 1 diabetes to other dietary exposures in infants and young children, including cereals with and without gluten, root vegetables, and fruit.

“I’m not sure there’s any one factor in the infant diet that we can say is most important,” Norris says. “There’s too much variability in what the studies show; it might not be any one factor.” She says data also suggest type 1 diabetes may be linked to enteroviruses^25^ and to pollutants including nitrates and nitrate-derived nitrosamines in drinking water.^26^

## Other Exposures Provide Clues to Mechanisms of Autoimmunity

Industrial compounds and chemicals are linked to autoimmune disease mainly by occupational studies, in which exposures are more reliably ascertained from memory and workplace records than they are in studies of the general population. Some of the best supported associations, Miller says, link occupational exposure to crystalline silica with illnesses such as RA, SLE, and systemic sclerosis (also known as scleroderma), a disease of the connective tissues.^27^,^28^,^29^

Jean Pfau, an associate professor of immunotoxicology at Idaho State University, suggests that silica and asbestos—which has been associated with RA, SLE, and scleroderma in miners and other residents in the former asbestos mining town of Libby, Montana^30^—evoke autoimmune disease in similar ways. Both compounds embed in the lungs, she says, which serves to attract immune cells and to produce inflammation. Moreover, silica and asbestos are cytotoxic, so they kill cells in ways that can generate a lot of cellular debris. What’s possible, Pfau says, is that B cells in the inflamed area might “lose tolerance” to self material—i.e., cell debris—and then go on to produce autoantibodies matched to that debris that target healthy cells throughout the body. Similar processes might be triggered by viral exposure, she says, for instance, to Epstein-Barr virus, which has been implicated in RA and SLE.^31^

Also important in the immunotoxicology literature is the solvent trichloroethylene (TCE), a ubiquitous groundwater contaminant. In a 2006 study by Gilbert and colleagues, TCE exposure altered the expression of T helper cells in mice, making them less susceptible to apoptosis (programmed cell death).^32^ “Apoptosis is supposed to prevent autoreactive T helper cells—or more specifically, the CD4^+^ T cell subset that expresses CD4 protein on its surfaces—from expanding and causing autoimmune disease,” Gilbert explains. “And so suppressing this process can enhance vulnerability to a range of different illnesses.”

Gilbert’s TCE-exposed mice showed evidence of immune activity in the liver—including cytokine alterations and changes in lymphocyte gene expression—at nontoxic doses.^32^ The mice eventually developed autoimmune hepatitis, she says, but TCE’s effects on CD4^+^ T cells aren’t necessarily limited to liver disease.

Meanwhile, a number of studies also link solvents, including TCE, to autoimmune illnesses such as RA, SLE, and systemic sclerosis.^27^ Whether those associations are real is difficult to tell, Gilbert says, because humans are typically exposed to chemical mixtures.

Pollard says well-controlled human studies are a missing link in autoimmune disease research. “These illnesses are rare, and so you need large studies to detect associations, and that costs a lot of money,” he says. “We have all these bits and pieces—for instance, various reports of people exposed in mines who have some features of autoimmune disease, but these people are also exposed to so much else. That’s the biggest problem: we need hard data on populations who are exposed and who are not exposed, and those studies aren’t easy to do.”

## Needs for the Future

What’s needed most, Miller says, are better data documenting the frequency and location of autoimmune diseases in the population. “We’re talking about a national registry, something that would allow us to get a handle on disease hot spots in relation to certain environmental exposures,” he says. “With that, we’d also be able to see how these illnesses are changing over time.”

Miller points to the National Cancer Institute’s Surveillance Epidemiology and End Results (SEER) program^33^ as an example of a successful registry. SEER collects information on cancer incidence, prevalence, and survival from areas representing 28% of the U.S. population and compiles trend data for the entire country. “We don’t have anything like that for autoimmune disease,” Miller says. The consequence, he adds, is that whereas cancers are often addressed as a single entity, autoimmune illnesses are put in disease-specific silos, which prevents more efficient use of research dollars.

According to Rose, better diagnostics also are a major priority. Today, he says most people with autoimmune diseases aren’t diagnosed until it’s already late in the disease process. “We have good evidence that these illnesses can go on for years before they become clinically evident,” Rose says. “By the time we’re seeing these patients, a lot of damage has already occurred, and we’re left with the difficult job of trying to fix it. It would be better to find them earlier, so better biomarkers that predict who’s at risk are an absolute necessity.”

## Figures and Tables

**Figure:**
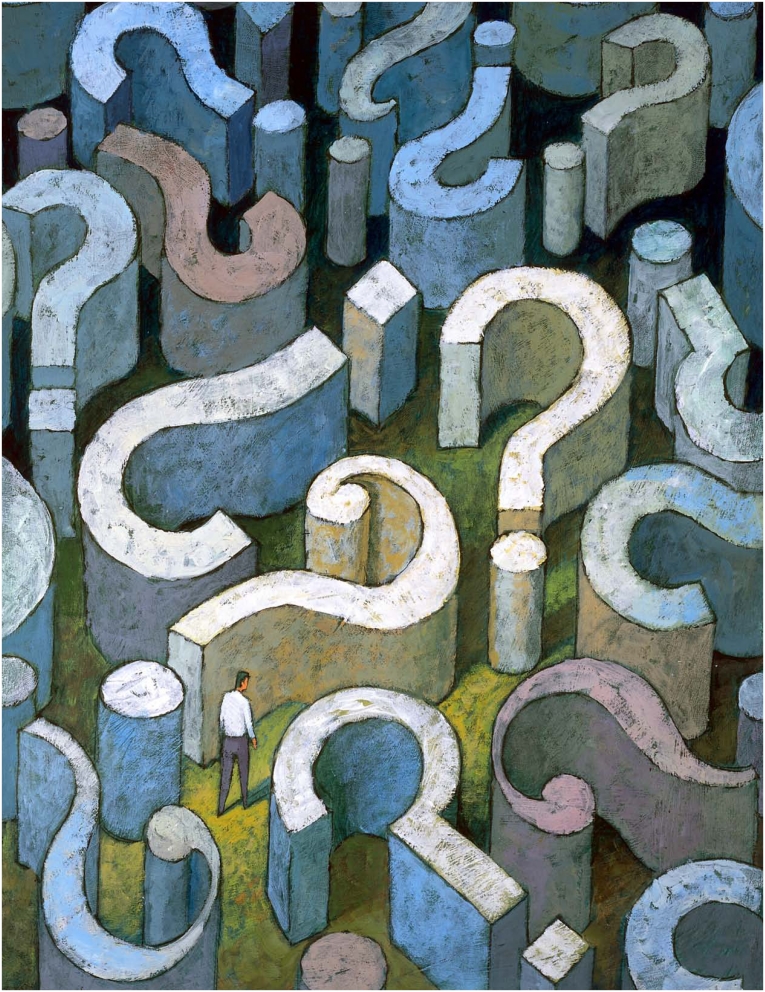


**Figure:**
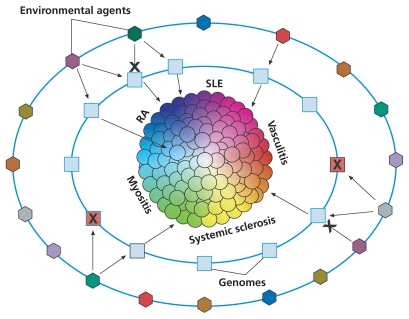
Gene–Environment Interactions and Autoimmune Disease: One Hypothesis In the elemental disorder hypothesis, autoimmune diseases are viewed as collections of many individual phenotypes, each defined by a unique set of symptoms, signs, and laboratory findings. This figure uses the example of systemic rheumatic diseases, a subset of autoimmune diseases, to conceptualize how a variety of disease phenotypes may result from different gene–environment interactions. Each sphere represents a disease phenotype, each square represents an individual’s genome, and each hexagon represents a particular environmental exposure. In this hypothesis, some combinations of genomes and environmental exposures lead to certain disease phenotypes, whereas other combinations might not. In still other cases, either an environmental factor or a genome may confer protection against developing disease, indicated here by an X. RA = rheumatoid arthritis; SLE = systemic lupus erythematosus. Source: Gourley M, Miller FW. Mechanisms of disease: environmental factors in the pathogenesis of rheumatic disease. Nat Clin Pract Rheumatol 3(3):172–180 (2007); doi:10.1038/ncprheum0435. Reprinted with permission.

**Figure:**
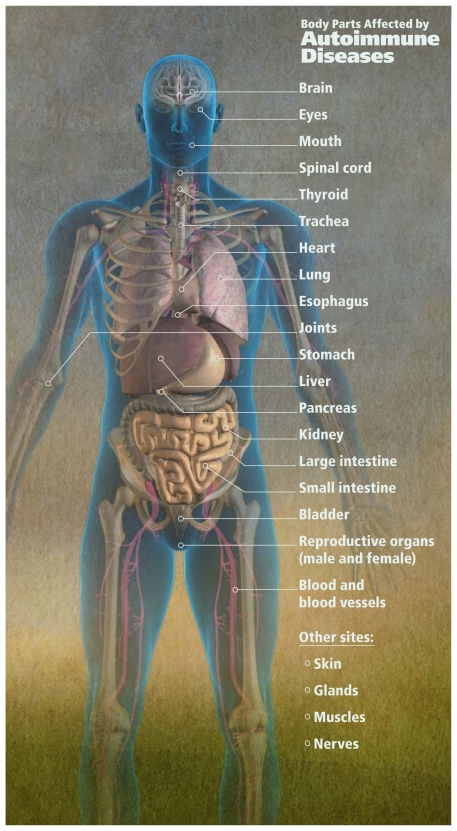
Autoimmune diseases are split into organ-specific and systemic varieties. Some sources cite as many as 120 distinct autoimmune diseases, which as a group are thought to affect 50 million Americans.

## References

[1] Segal JB, Powe NR (2006). Prevalence of immune thrombocytopenia: analyses of administrative data. J Thrombo Haemost.

[2] Jacobson DL (1997). Epidemiology and estimated population burden of selected autoimmune diseases in the United States. Clin Immunol Immunopathol.

[3] The Autoimmune Diseases Coordinating Committee (2005). Progress in Autoimmune Disease Research. Report to Congress. NIH Publication 05-5140.

[4] AARDA (2011). The Cost Burden of Autoimmune Disease: The Latest Front in the War on Healthcare Spending.

[5] AARDA Questions & Answers. How Many Americans Have an Autoimmune Disease? [website].

[6] Podar T (2001). Increasing incidence of childhood-onset type I diabetes in 3 Baltic countries and Finland 1983–1998. Diabetologia.

[7] Grimaldi LME (2007). High prevalence and fast rising incidence of multiple sclerosis in Caltanissetta, Sicily, Southern Italy. Neuroepidemiology.

[8] Rubio-Tapia A (2009). Increased prevalence and mortality in undiagnosed celiac disease. Gastroenterology.

[9] Pollard KM (2010). Toxicology of autoimmune diseases. Chem Res Toxicol.

[10] Nagata C (1995). Systemic lupus erythematosus: a case–control epidemiologic study in Japan. Int J Dermatol.

[11] Hardy CJ (1998). Smoking history, alcohol consumption, and systemic lupus erythematosus: a case–control study. Ann Rheum Dis.

[12] Bengtsson AA (2002). Risk factors for developing systemic lupus erythematosus: a case–control study in southern Sweden. Rheumatology.

[13] Weetman AP (2001). Determinants of autoimmune thyroid disease. Nature Immunol.

[14] Vestergaard P (2002). Smoking as a risk factor for Graves’ disease, toxic nodular goiter, and autoimmune hypothyroidism. Thyroid.

[15] Reidenberg MM (1993). Acetylation phenotypes and environmental chemical exposure of people with idiopathic systemic lupus erythematosus. Arthritis Rheum.

[16] Harjutsalo V (2008). Time trends in the incidence of type 1 diabetes in Finnish children: a cohort study. Lancet.

[17] Patterson CC (2009). Incidence trends for childhood type 1 diabetes in Europe during 1989–2003 and predicted new cases 2005–20: a multicentre prospective registration study. Lancet.

[18] (2010). SEARCH for Diabetes in Youth Study [website].

[19] Lamb MM (2009). Height growth velocity, islet autoimmunity, and type 1 diabetes development: the Diabetes Autoimmunity Study in the Young. Diabetologia.

[20] Knip M (2010). Infant feeding and the risk of type 1 diabetes. Am J Clin Nutr.

[21] Ziegler A-G (2003). Early infant feeding and risk of developing type 1 diabetes–associated autoantibodies. JAMA.

[22] Virtanen SM (2006). Age at introduction of new foods and advanced beta cell autoimmunity in young children with HLA-conferred susceptibility to type 1 diabetes. Diabetologia.

[23] Norris JM (2003). Timing of initial cereal exposure in infancy and risk of islet autoimmunity. JAMA.

[24] Couper JJ (1999). Lack of association between duration of breast-feeding or introduction of cow’s milk and development of islet autoimmunity. Diabetes.

[25] Yeung WC Enterovirus infection and type 1 diabetes mellitus: systematic review and meta-analysis of observational molecular studies. BMJ.

[26] Longnecker MP, Daniels JL (2001). Environ Health Perspect.

[27] Gourley M, Miller FW (2007). Mechanisms of disease: environmental factors in the pathogenesis of rheumatic disease. Nat Clin Practice Rheumatol.

[28] Calvert GM (2003). Occupational silica exposure and risk of various diseases: an analysis using death certificates from 27 states of the United States. Occup Environ Med.

[29] Stolt P (2003). Quantification of the influence of cigarette smoking on rheumatoid arthritis: results from a population based case–control study, using incident cases. Ann Rheum Dis.

[30] Noonan CW (2006). Nested case–control study of autoimmune disease in an asbestos-exposed population. Environ Health Perspect.

[31] Costenbader KH, Karlson EW (2006). Epstein–Barr virus and rheumatoid arthritis: is there a link?. Arthritis Res Ther.

[32] Gilbert KM (2006). Environmental contaminant trichloroethylene promotes autoimmune disease and inhibits T-cell apoptosis in MRL^+/+^ mice. J Immunotoxicol.

[33] Surveillance Epidemiology and End Results (SEER) Program [website].

